# The Impact of Gut Microbiota-Derived Metabolites in Autism Spectrum Disorders

**DOI:** 10.3390/ijms221810052

**Published:** 2021-09-17

**Authors:** Lucía N. Peralta-Marzal, Naika Prince, Djordje Bajic, Léa Roussin, Laurent Naudon, Sylvie Rabot, Johan Garssen, Aletta D. Kraneveld, Paula Perez-Pardo

**Affiliations:** 1 Division of Pharmacology, Utrecht Institute for Pharmaceutical Sciences, Faculty of Science, Utrecht University, 3584 CG Utrecht, The Netherlands; n.z.prince@uu.nl (N.P.); j.garssen@uu.nl (J.G.); A.D.Kraneveld@uu.nl (A.D.K.); 2 Department of Ecology and Evolutionary Biology, Yale University, New Haven, CT 06511, USA; djordje.bajic@yale.edu; 3 Microbial Sciences Institute, Yale University, West Haven, CT 06516, USA; 4 Micalis Institute, INRAE, AgroParisTech, Université Paris-Saclay, 78350 Jouy-en-Josas, France; lea.roussin@inrae.fr (L.R.); sylvie.rabot@inrae.fr (S.R.); 5 CNRS, Micalis Institute, INRAE, AgroParisTech, Université Paris-Saclay, 78350 Jouy-en-Josas, France; laurent.naudon@inrae.fr; 6 Danone Nutricia Research, 3584 CT Utrecht, The Netherlands

**Keywords:** autism spectrum disorders, microbiota–gut–brain axis, bacterial metabolites

## Abstract

Autism Spectrum Disorder (ASD) is a set of neurodevelopmental disorders characterised by behavioural impairment and deficiencies in social interaction and communication. A recent study estimated that 1 in 89 children have developed some form of ASD in European countries. Moreover, there is no specific treatment and since ASD is not a single clinical entity, the identification of molecular biomarkers for diagnosis remains challenging. Besides behavioural deficiencies, individuals with ASD often develop comorbid medical conditions including intestinal problems, which may reflect aberrations in the bidirectional communication between the brain and the gut. The impact of faecal microbial composition in brain development and behavioural functions has been repeatedly linked to ASD, as well as changes in the metabolic profile of individuals affected by ASD. Since metabolism is one of the major drivers of microbiome–host interactions, this review aims to report emerging literature showing shifts in gut microbiota metabolic function in ASD. Additionally, we discuss how these changes may be involved in and/or perpetuate ASD pathology. These valuable insights can help us to better comprehend ASD pathogenesis and may provide relevant biomarkers for improving diagnosis and identifying new therapeutic targets.

## 1. Introduction

A vast number of microbes including bacteria, fungi, archaea and viruses coexist in the human body. These microorganisms mostly colonise the intestinal tract and are known as the gut microbiota [1]. Among individuals, microbial communities are very diverse and have a wide range of genetic capacities [2]. There is a growing interest in how these complex and dynamic microbial communities colonising the intestine influence health and disease. It has been shown that a balanced relationship between gut microbiota and the host is essential to maintain gut homeostasis as well as the host’s health. By contrast, an imbalance in the gut’s microbial community, also referred to as gut dysbiosis, may lead to disease development [3]. Multiple studies have reported gut dysbiosis in (i) intestinal disorders such as Crohn’s disease and ulcerative colitis [4], (ii) metabolic disorders such as diabetes and obesity [5,6], and (iii) neurological disorders such as Alzheimer’s disease and Autism Spectrum Disorder (ASD) [7,8]. 

Autism Spectrum Disorders are a set of neurodevelopmental disorders diagnosed early in childhood. However, they are often misdiagnosed or undiagnosed due to the subjectivity of the diagnostic methodologies, the heterogeneity of symptoms, and the lack of specific biomarkers [9]. The diagnosis is based on behavioural characteristics such as deficiencies in social interaction and communication [10]. Epidemiological studies on ASD prevalence show highly variable results, although the increasing trend of cases in recent years is indisputable [11]. The worldwide estimation of ASD prevalence ranges from 1.76/1000 to 7.17/1000 [12]. There is no standard treatment procedure for autistic populations. Children suffering from ASD receive different treatments, including educational interventions and medicines that try to improve their quality of life by relieving disease-related symptoms such as depression, insomnia, and trouble focusing [13]. This lifelong therapy and the disorder per se lead to an economic and physiological burden to the individual and to the families. Moreover, the lack of a specific treatment complicates the integration of individuals with ASD in society. 

Individuals affected by ASD are a heterogeneous group with differences in symptoms and prevalence of developing comorbid medical conditions. However, intestinal problems are often present in these patients, which may reflect aberrations in the bidirectional communication between the brain and the gut [14,15]. Furthermore, children with ASD suffering from gastrointestinal complaints exhibit an enhanced behavioural dysfunction, such as anxiety and social withdrawal, compared to autistic children without intestine-related symptoms [16,17,18]. Recent reports described the impact of gut microbiota in brain development and behavioural function [19]. In addition, changes in the gut microbiota composition were repeatedly reported in ASD individuals when compared to typically developed children [7,15,17]. While correlation does not equate to causation, changes in the gut microbiota composition and, more importantly, its function can be involved in or exacerbate the development of ASD phenotype. This review aims to (i) report emerging literature showing changes in microbiota composition and metabolite production of ASD individuals compared to healthy controls, and (ii) discuss how these changes may contribute and/or perpetuate ASD pathology. These valuable insights can help us to better comprehend the pathogenesis and may provide relevant biomarkers for better diagnosis and for the identification of therapeutic targets.

## 2. Methodology

The identification of potentially relevant articles for this review was conducted using three electronic databases (PubMed, Google Scholar and Scopus). Our search strategy included a combination of keywords and MESH terms together with “AND” or “OR”. Several key terms that were employed include Autism Spectrum Disorder, ASD, metabolism, metabolites, microbial metabolites, gut bacteria, microbiota, microbiota composition, human study, animal study, among others. Over a hundred articles, published over the years, were reviewed. The final selection includes articles published between 2002 and 2020 that are presented in Table 1 and Table 2, Tables S1 and S2. We excluded: (i) studies not in English, (ii) reviews and case reports, (iii) in vitro studies, and (iv) studies lacking sufficient information about the studied groups (e.g., age, sex, and methodology of ASD diagnosis). We included twenty-two articles (seventeen human studies and five animal studies) for changes in gut microbiota composition in ASD (Table 1 and Table S1) and twenty-seven paired-matched studies that compare ASD individuals to control individuals regarding metabolic profile (Table 2). In addition, we selected eight studies that investigated metabolic changes in animal models for ASD (Supplementary Table S2).

## 3. Interplay between Microbiota, Gut and Brain

The interaction between brain and intestine, often referred to as the “gut-brain axis”, is highly complex and potentially involves multiple (and parallel) communication pathways, including roles for the immune and entero-endocrine system, but also metabolic and neuronal communication via the enteric nervous system (ENS) [20]. Moreover, the intertwined relationship between gut microbiota and intestinal homeostasis is a contributing factor in brain development and behaviour [21,22]. Microbial dysbiosis in the gut has been widely related to several neurological diseases such as ASD, indicating its possible influence on the pathophysiology [23]. 

Recent studies have shown the impact of the gut microbiota in brain development, behavioural function as well as blood–brain barrier (BBB) integrity [7,18,21,24,25,26]. Intestinal dysbiosis early in life may lead to neurodevelopmental disturbances. Previous evidence indicates that a dysfunctional intestinal tract is associated with impaired gut barrier integrity (also referred to as “leaky gut”), allowing the entry of bacteria and/or bacterial-derived products into the systemic circulation, which in turn may cross the BBB leading to neural dysfunction. In ASD, the microbiota can exert its influence on neural function by causing low-grade systemic inflammation, abnormal serotonergic system, and/or altered metabolic profile [27]. However, the causal relationship of altered gut microbiota composition with ASD pathogenesis still needs to be defined. 

In previous years, the main pathways that describe the relationship between microbiota, gut and the nervous system were thoroughly reviewed [7,8,20,25,28,29,30,31,32]. First, intestinal microbes can modulate brain function by directly affecting the enteric nervous system, and by producing bioactive metabolites in the gut such as neurotoxins and neurotransmitters [20]. For example, specific bacteria were reported to produce neuroactive molecules, such as GABA (γ-aminobutyric acid; an inhibitory neurotransmitter), that modulate specific neuronal responses as pain perception [33]. Previous results suggest that the communication between gut microbiota-induced changes in the GABAergic system and brain function is mediated via the vagal nerve [34]. Second, the gut microbiota is known to affect the endocrine system such as 5-hydroxytryptamine (5-HT; serotonin, a neurotransmitter controlling multiple functions in intestine and brain) production and hypothalamic–pituitary–adrenal (HPA) axis function. Microbes influence, both directly and indirectly, the production of biologically active molecules by the host affecting gut and brain function [30,31]. For example, impaired HPA axis together with compromised gut bacterial composition were linked to behavioural problems such as anxiety and depression [35]. Third, intestinal homeostasis is critical for the proper function of the immune system. Most of the immune cells present in our body find their origin in the gut, thus intestinal dysbiosis may lead to both local and systemic immune dysregulation. Last, the complex bacterial network in our gut has a great impact on the host’s metabolism. The genetic capacity of gut microbiota greatly exceeds ours, which means that bacterial communities colonising the human gut have large metabolic activities. Abnormal amounts of certain bacterial-produced metabolites can lead to neuroimmune complications [36]. There are multiple pieces of evidence supporting the implication of intestinal microbes in the gut–brain axis [8], however, the exact molecular mechanisms remain unclear. Revealing how microbiota, gut and brain communicate is crucial to understand their implication in ASD and other neurological diseases. This review specifically focuses on the metabolic pathways since their study may contribute to a better understanding of the metabolic aspect of the disease.

## 4. ASD-Associated Differences in Faecal Bacterial Composition

In a healthy state, a balanced relationship exists between bacterial communities that colonise the gut and other organs of the host [1]. Dysbiotic faecal microbial compositions have been repeatedly described in individuals affected by ASD. Despite that the main aim of this review is to gather and discuss reported metabolic changes mediated by gut microbiota in individuals with ASD, we also summarise part of the most relevant data about faecal microbiota composition in ASD (Table 1 and Table S1). 

Highly diverse gut microbiota is thought to promote the host’s health [37]. Conversely, less diverse microbial communities in the intestine have been frequently reported in several neurological disorders [28,38] including ASD [39,40,41,42,43]. Specifically, Dan et al. (2020) investigated microbial diversity in children with ASD, but only the subgroup characterised by ASD core symptoms together with constipation had significantly decreased microbial diversity compared to healthy children [43]. Such results were also consistently shown in different murine models of ASD [44,45,46]. In addition, opposite data or no significant differences in bacterial diversity were also described in other studies [47,48,49]. All this together suggests that less diverse gut microbiota might be characteristic of a subset of individuals diagnosed with ASD, also affected by intestinal abnormalities.

Bacteroidetes and Firmicutes are two phyla that compose around 90% of the total bacterial species present in the human gut [50]. Perturbations in the abundance of these dominant phyla were associated with ASD [43,51,52,53]. Studies of the microbial composition of ASD children at the phylum level particularly revealed a higher amount of Bacteroidetes and a lower abundance of Firmicutes compared to healthy children [47,48,49,53,54]. Nonetheless, not all studies are in line with this result [52,55]. Overall, these data suggest that abnormal levels of gut bacterial communities at the phylum level are related to ASD, while deeper studies exploring species levels may give us more detailed insights about specific clinical effects. 

At the genus level, multiple bacterial abundances have shown discrepancies when comparing individuals diagnosed with ASD to healthy children (Table 1). Lower proportions of *Bifidobacterium* (lactic acid bacteria, considered to be beneficial bacteria for the host), *Prevotella* (bacteria associated with high fibre diets, hypothesised to be beneficial for the host) and *Akkermansia* (besides being mucin-degrading bacteria, it has been repeatedly shown to provide health-promoting effects) were reported to be associated with ASD. On the other hand, the presence of specific *Clostridia* species (often known as pathobiont bacteria, potentially disease-causing microorganisms) was also reported to be linked to ASD [17,40,41,42,47,48,49,54,55,56,57]. Considering all these lines of associative evidence, gut dysbiosis has drawn scientists’ attention, and it is a major area of study in ASD and other neurodevelopmental disorders. Lack of consensus in the studies describing gut microbiota diversity in ASD may be explained by small sample sizes, age variability, and difficult standardisation of lifestyle among the subjects of the studies in addition to sampling processing and platforms used for microbiomics.

**Table 1:** Differences in faecal microbiota composition in ASD.

	ASD	
	Increased	Decreased
α-diversity	[47,48,49]	[40,41,42,43,44,45,46]
Bacteroidetes/Firmicutes	[53]	[43,51,52]
Firmicutes	[54,55,58]	[44,47,48]
* Blautia*	-	[44,49,59]
* Clostridium*	[42,47,48,56,58,59,60]	[47]
* Coprococcus*	-	[40,44,48,49]
* Dorea*	[45,48,52]	[59]
* Enterococcus*	[45]	[17,55]
* Faecalibacterium*	[49]	[41,45,48]
* Lactobacillus*	[17,51,52,55]	-
* Roseburia*	[49,60]	-
* Ruminococcus*	[49]	[43,47,48,60]
* Streptococcus*	-	[47,49,53]
Bacteroidetes	[44,47,49,53,54]	[52,58]
* Bacteroides*	[44,47,48,49]	[42,43,46]
* Prevotella*	[54,60]	[40,41,43,45,48,55]
Proteobacteria	[47,49]	[54]
* Desulfovibrio*	[45,47]	[43,44,55]
* Escherichia*	[43]	[48,53]
* Klebsiella*	[42]	[17]
* Parasutterella*	[48]	[58]
* Shigella*	[43]	[48]
* Sutterella*	-	[43,59]
Actinobacteria	-	[47,49,54]
* Bifidobacterium*	[43,55]	[17,44,47,48,49,57]
Verrucomicrobia	-	[54]
* Akkermansia*	[40,44,48,54]	[57]

This table sums up data reported in human and animal studies for ASD regarding (i) α-diversity, (ii) Bacteroidetes/Firmicutes ratio and (iii) bacterial abundances at phylum and genus levels. See Supplementary Table S1 for more details.

## 5. ASD-Associated Differences in Gut Microbial Metabolites

Gut microbes have a vast genome, thus metabolic functions, which makes them key modulators of health and disease [61]. It is currently known that microbial-derived metabolites are involved in immune, metabolic, neurological and gastrointestinal clinical conditions [62,63]. In the past fifteen years, the metabolic profile of individuals affected by ASD was investigated in a more extensive manner. Results are summarised in Table 2, indicating significant dissimilarities in samples obtained from blood, urine, faeces, brain and intestinal tissue when comparing healthy individuals to people diagnosed with ASD. Additionally, metabolic differences associated with ASD reported in animal studies are summarised in Supplementary Table S2.

**Table 2:** Metabolic differences associated with gut microbiota in individuals affected by ASD.

	Study Group					Metabolic Assessment			
Reference	Country	Sample Size	Male:Female	Mean Age	Associated Symptoms	Sample Type	Method	Results	Molecular Pathways
[64]	USA	20 ASD patients 33 control individuals	Unspecified	Unspecified	-	Plasma	HPLC	**ASD patients vs. Controls** ↓ _total_GSH, _total_GSH/GSSG, SAM/SAH	Oxidative stress and methylation dysfunction
[65]	USA	262 ASD patients 60 control individuals	211:51 30:30	2–13 yo 2–13 yo	-	Urine	GC+MS	**ASD patients vs. Controls** ↑ HPHPA	Altered gut bacterial metabolism (AAA)
[66]	Australia	39 ASD patients 28 neurotypical siblings 34 control individuals	35:4 14:14 17:17	3–9 yo	-	Urine	MRS	**ASD patients vs. Controls** ↑ Taurine, succinate, acetate, dimethyl amine, N-methyl nicotinic acid and N-methyl nicotinamide ↓ Glutamate, hippurate, phenylacetylglutamine	Oxidative stress, altered AA, nicotinic and gut bacterial metabolism (AAA)
[17]	USA	58 ASD patients 39 control individuals	50:8 18:21	6.91 ± 3.4 yo	Gastrointestinal problems	Faeces	GC+FID	**ASD patients vs. Controls** ↓ Acetate, valerate, propionate and butyrate	Altered gut bacterial metabolism (SCFAs)
[67]	Italy	59 ASD individuals 59 control individuals	44:15 44:15	8.29 ± 0.56 yo 8.46 ± 0.59 yo	-	Urine	HPLC+UV	**ASD patients vs. Controls** ↑ p-cresol	Altered gut bacterial metabolism (AAA)
[68]	USA	48 ASD patients 53 control individuals	36:12 34:19	10.7 ± 4.0 yo 10.2 ± 3.8 yo	Gastrointestinal problems	Urine	LC/GC+MS	**ASD patients vs. Controls** ↑ 2-(4-hydroxyphenyl) propionate and taurocholonate sulfate ↓ free AA and carnosine	Oxidative stress, altered gut bacterial metabolism (AA)
[69]	USA	27 ASD patients 27 control individuals	Unspecified	Unspecified	Oxidative stress	Cerebellum Temporal cortex	HPLC+MS	**ASD patients vs. Controls** ↑ 3-nitrotyrosine, 3-chlorotyrosine and 8-oxo-deoxyguanosine ↓ GSH and GSH/GSSG	Oxidative stress and mitochondrial dysfunction
[70]	China	23 ASD individuals 31 control individuals	21:2 15:16	123 ± 9 mo 136 ± 9 mo	-	Faeces	HPLC GC+MS	**ASD patients vs. Controls** ↑ total SCFAs, acetic acid, propionic acid, butyric acid, isobutyric acid, valeric acid ammonia	Altered gut bacterial metabolism (SCFAs)
[71]	France	26 ASD individuals 24 control individuals	22:4 16:8	6–9 yo 6–9 yo	-	Urine	GC+MS	**ASD patients vs. Controls** ↑ Succinate, Glycolate ↓ Hippurate, 3-hydroxyhippurate, 3-hydroxyphenylacetate, indole-3-acetate, phosphate	Altered gut bacterial metabolism (AA)
[72]	USA	18 ASD + MD + patients 18 ASD + MD – patients 54 control individuals	14:4 15:3 Unspecified	8.5 ± 3 yo 7.9 ± 3.2 yo Unspecified	Mitochondrial disease (MD)	Plasma	HPLC	**ASD + MD + patients vs. ASD + MD-** ↑ GSSG and _free_GSH/GSSG **Both ASD groups vs. Controls** ↓ _free_GSH and _free_GSH/GSSG ↑ 3-clorotyrosine	Oxidative stress and mitochondrial dysfunction
[73]	France	33 ASD patients 33 control individuals	29:4 29:4	7.9 ± 0.57 yo 7.6 ± 0.61 yo	-	Urine	HPLC	**ASD patients vs. Controls** ↑ p-cresol, p-cresylsulfate, p-cresylglucuronate	Altered gut bacterial metabolism (AAA)
[74]	USA	52 ASD individuals 30 control individuals	41:11 26:4	5.37 ± 0.81 yo 5.6 ± 0.95 yo	-	Plasma	LC/GC+MS LC+HMRS+MS	**ASD patients vs. Controls** ↑ Aspartic acid, serine, glutamic acid, glutaric acid, succinic acid, 3-aminoisobutyric acid ↓ Homocitrulline, 2-hydroxyvaleric acid, cystine, isoleucine, creatinine, 4-hydroxyphenyllactic acid, citric acid, lactic acid, heptadecanoic acid, myristic acid	Altered energy metabolism, mitochondrial dysfunction and oxidative stress
[75]	Australia	15 ASD individuals 12 control individuals	10:5 10:2	8.47 ± 2.36 yo 9.61 ± 2.9 yo	-	Serum	UPLC+FLD GC+MS	**ASD patients vs. Controls** ↑ Kynurenine/tryptophan, kynurenine, quinolinic acid ↓ picolinic acid	Altered kynurenine pathway
[76]	China	73 ASD individuals 63 control individuals	59:14 51:12	4.6 ± 0.8 yo 4.1 ± 0.7 yo	-	Serum	UPLC+ Q-TOF+MS	**ASD patients vs. Controls** ↑ Phytosphingosine, pregnanetriol, lysophosphatidylcholines, lysophosphatidylethanolamines, sphingosine 1-phosphate ↓ L-acetylcarnitine, uric acid, decanoylcarnitine, arachidonic acid, docosahexaenoic acid, adrenic acid, docosapentaenoic acid	Altered fatty acid metabolism, mitochondrial dysfunction and immune dysregulation
[77]	China	62 ASD individuals 62 control individuals	48:14 48:14	3.69 ± 1.62 yo 3.45 ± 1.62 yo	-	Urine	GC+MS	**ASD patients vs. Controls** ↑ HPHPA, 3-hydroxyphenylacetic acid and 3-hydroxyhippuric acid	Altered gut bacterial metabolism (AAA)
[49]	Italy	11 ASD patients 14 control individuals	9:2 8:6	35 ± 5.7 mo 35 ± 8.4 mo	Gastrointestinal problems	Faeces	GC+MS	**ASD patients vs. Controls** ↑ Butyrate	Altered gut bacterial metabolism (SCFAs)
[41]	USA	21 ASD patients 23 control individuals	15:6 22:1	10.1 ± 4.1 yo 8.4 ± 3.4 yo	Gastrointestinal problems	Faeces	(H) MRS	**ASD patients vs. Controls** ↓ GABA, lactate, butyrate, acetate, propionate, formate, nicotinate, glutamate, aspartate ↑ tyrosine, p-cresol and isopropanol	Altered neurotransmitter and gut bacterial metabolism (AAA, SCFAs)
[78]	China	60 ASD individuals 30 control individuals	49:11 25:5	42.86 ± 11 mo 39.3 ± 12.9 mo	-	Serum	MS/MS	**ASD patients vs. Controls** ↓ free carnitine, glutaryl carnitine, octyl carnitine, 24 carbonyl carnitine, carnosyl carnitine	Altered fatty acid metabolism, mitochondrial dysfunction
[79]	Russia	32 ASD patients 40 control individuals	23:9 27:13	2–60 yo 1–62 yo	-	Prefrontal cortex	LC+MS	**ASD patients vs. Controls** the concentrations of 205 out of 1366 analysed metabolites showed significant differences	Altered metabolisms: glutathione, purine, pyruvate, propanoate, TCA cycle, galactose, starch and sucrose, nicotinate and nicotinamide, cysteine and methionine, and arginine and proline
[42]	China	43 ASD individuals 31 control individuals	36:7 17:14	4.51 ± 2.23 yo 3.14 ± 1.73 yo	Gastrointestinal problems	Faeces	LC+MS	**ASD patients vs. Controls** ↑ Taurocholic acid ↓ 2-keto-glutaramic acid, L-aspartic acid, L-phenylalanine, L-tyrosine, epinephrine, cortisol	Altered neurotransmitter and gut bacterial metabolism (AA: glutamate and tyrosine)
[43]	China	143 ASD patients 143 control individuals	130:13 127:16	4.9 ± 0.16 yo 5.2 ± 0.17 yo	Gastrointestinal problems	Faeces	LC+MS	**ASD patients vs. Controls** 37 metabolites showed significant differences	Altered fatty acid, purine and pyrimidine, neurotransmitter and gut bacterial metabolism (AA)
[80]	Italy and Northern Europe	40 ASD patients 40 control individuals	31:9 29:11	4.95 ± 0.45 yo 4.35 ± 0.55 yo	-	Urine	UHPLC+MS	**ASD patients vs. Controls** ↑ p-cresol, ascorbate, dopamine, homovanillic aid, glutamate ↓ norafrenaline, adrenaline, vanillylmanelic acid, GABA, pyridoxal phosphate	Altered neurotransmitter and gut bacterial metabolism (AA)
[81]	Japan	98 ASD individuals 77 control individuals	73:25 39:38	7.08 ± 2.87 yo 8.49 ± 3.75 yo	Insomnia, depression and anxiety	Plasma	d-ROMs test BAP test	**ASD patients vs. Controls** ↑ dROM ↓ BAP/dROM	Oxidative stress and compromised antioxidant capacity
[82]	Slovakia	24 ASD individuals 13 control individuals	24:0 13:0	7.7 ± 0.9 yo 8.2 ± 1.2 yo	-	Urine	UHPLC+MS	**ASD patients vs. Controls** ↑ Indoxyl sulfate	Altered gut bacterial metabolism (AAA)
[83]	China	164 ASD patients 164 control individuals	129:35 129:35	5 ± 1.0 yo 5 ± 1.0 yo	-	Plasma	HPLC+MS	**ASD patients vs. Controls** ↑ TMAO, choline	Altered gut bacterial metabolism (choline)
[84]	Japan	30 ASD patients 30 control individuals	25:5 17:13	8.27 ± 1.21 yo 7.96 ± 1.44 yo	-	Plasma	CE+MS	**ASD patients vs. Controls** ↑ 48 significant metabolites showed significant differences	Altered lipid biosynthesis and metabolism, oxidative stress and synaptic function

Summary of twenty-seven case-control studies conducted in the last fifteen years. The studies are presented based on the year of publication from less to more recent. ASD: autism spectrum disorder, mo: months old, yo: years old, MD: mitochondrial disease, BAP: biological antioxidant potential, dROM: reactive oxygen metabolites, FID: flame ionisation detector, FLD: fluorescence detector, GC: gas chromatography, HPLC: high-performance liquid chromatography, LC: liquid chromatography, (H) MRS: (proton) magnetic resonance spectroscopy, MS: mass spectrometry, Q-TOF: quadrupole-time-of-flight, UHPLC: ultra-high-performance liquid chromatography, UPLC: ultraperformance liquid chromatography, UV: ultraviolet detector, (A) AA: (aromatic) amino acid, GSH: reduced glutathione, GSSG: oxidised glutathione, HPHPA: 3-(3-hydroxyphenyl)-3-hydroxypropionic acid, SAH: S-adenosyl-L-homocysteine, SAM: S-Adenosyl-L-methionine, SCFA: short-chain fatty acid, TMAO: trimethylamine N-oxide.

The interaction between human gut microbiota and host metabolic function has been extensively investigated, however, the exact mechanisms are not fully understood. A major limitation of this field of study is to differentiate between host and bacterial metabolism. Currently, in silico techniques that build metabolic networks to study the gut environment are becoming popular [85]. The metabolic pathways that were described to play a role in ASD are the following: (i) energy metabolism, (ii) protein and amino acid metabolism, (iii) lipid and fatty acid metabolism, and (iv) redox metabolism. Besides that, the microbiota plays an important role by being able to regulate intestinal and BBB integrity which may cause the translocation of certain metabolites from the intestinal lumen into the bloodstream eventually reaching the brain [86,87]. Additionally, the gut microbiota plays an important role in human physiology by forming a defence barrier for protection against pathogens and by stimulating the maturation of the immune system [88], as well as the development of behavioural and cognitive systems [19]. In addition, several bacteria colonising the human gut are able to synthesise neurotransmitters such as GABA, serotonin, dopamine, norepinephrine and acetylcholine [89].

**Figure 1:**
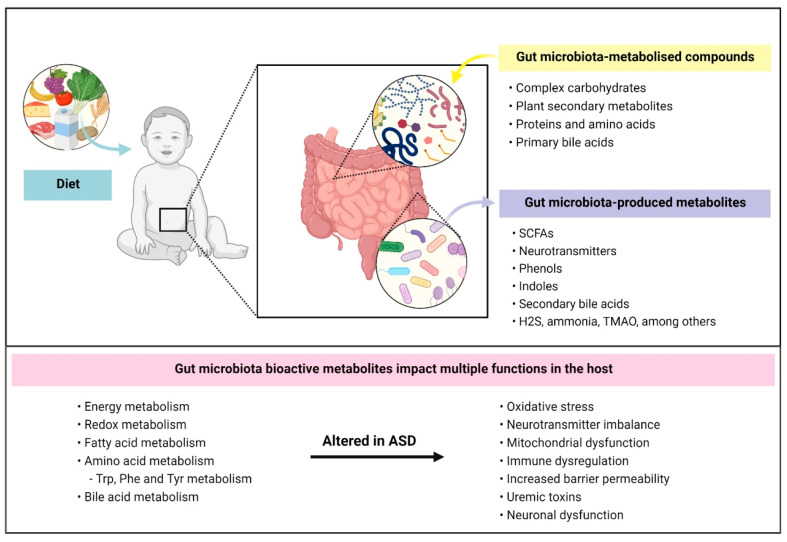
Gut-bacterial dependent metabolic pathways and their influence in ASD. Diet is a major contributing factor that influences gut microbiota composition and function. Intestinal bacteria mainly metabolise compounds that are unabsorbed by our gastrointestinal tract including (i) complex carbohydrates, (ii) plant secondary metabolites, (iii) proteins and amino acids, that come from digestive secretions, protein turnover and/or escape host digestion, and (iv) primary bile acids. Bacterial metabolisation of these compounds results in the production of bioactive metabolites such as short-chain fatty acids, neurotransmitters, indolic and phenolic compounds. Several metabolic pathways were reported to be altered in ASD such as redox and aromatic acid metabolism. In turn, metabolic dysfunction mediated by gut microbiota may lead to other complications in individuals affected by ASD such as impaired neuronal function, oxidative stress and increased intestinal and blood–brain barrier permeability. SCFAs: short-chain fatty acids; H2S: hydrogen sulfide; TMAO: trimethylamine N-oxide; Trp: tryptophan; Phe: phenylalanine; Tyr: Tyrosine.

Host metabolic changes influenced by gut bacteria in ASD have been linked to oxidative stress, mitochondrial dysfunction, genetic expression changes, immune dysfunction and neural abnormalities such as BBB permeability, microglia activation, altered neurotransmitter production and synaptic dysfunction (Figure 1). Investigating how bacterial communities interact with host metabolism can greatly contribute to better comprehend ASD pathophysiology.

### 5.1. Bacterial-Derived Metabolites from Complex Polysaccharide Metabolism

SCFAs, mainly acetate, propionate and butyrate, are by far the most investigated bacterial-produced metabolites involved in health and disease. These bioactive compounds influence the host in several ways; some of their biological activities are: (i) the source of energy for intestinal cells, (ii) the production of hormones that regulate hunger/satiety homeostasis, (iii) the modulation of the immune system, (iv) the stimulation of gut motility and epithelial barrier integrity, and (v) the impact on brain development and social behaviour [61,62,90,91,92,93]. Complex carbohydrates or dietary fibres that escape the host’s digestion are the main sources for the production of SCFAs by gut microbiota. It should be mentioned that SCFA production does not solely arise from the metabolic capacity of a specific bacterium, but also from their collaborative relationship [94]. Their presence has been broadly linked to exert health-promoting effects on the host, including immunomodulatory properties, intestinal barrier integrity maintenance and enteric nervous system stimulation, which controls functions such as intestinal peristalsis [95].

In ASD, SCFA analyses from urine, faecal and caecal samples have provided controversial results, thus their exact relation in the disease’s pathophysiology remains to be elucidated. Two studies showed lower quantities of acetate, propionate and butyrate measured in faeces from autistic children when compared to healthy controls [17,41]. On the other hand, several human and animal studies reported higher concentrations of the three aforementioned bacterial metabolites in both faecal and caecal samples from autistic groups when compared to control groups [44,48,49,58,70]. Although the blood concentrations are considerably lower compared to intestinal or faecal samples, systemic levels of SCFAs can be also measured in serum and plasma [96]. Investigating their systemic presence can contribute to understanding the connection of SCFA produced by gut microbiota with brain function. To our knowledge, SCFAs have not been explored in plasma or serum from individuals with ASD.

Acetate is the most abundant SCFA circulating in the colon and bloodstream and is produced by a vast number of anaerobic bacteria including some species from *Prevotella*, *Bifidobacterium* and *Ruminococcus* genera [97]. Although there are discrepancies in bacterial abundances in ASD, these three genera were widely reported to be decreased in individuals with ASD (Table 1). SCFAs were shown to promote intestinal mucosa homeostasis by stimulating immunoglobulin A (IgA) synthesis and reducing proinflammatory molecules such as tumour necrosis factor α (TNF-α) produced by immune cells. Acetate, together with propionate and butyrate, modulate the host’s immunity via the activation of different G-protein-coupled receptors (GPR41 and GPR43), which in turn trigger a protective immune response mediated by mitogen-activated kinase pathways [98]. In addition, circulating acetate can cross the BBB where it can serve as a carbon source being utilised by neural cells, and as a signalling compound modulating appetite and food intake [99]. Lower levels of acetate were observed in faecal and caecal samples from individuals with ASD [17,41,44]. On the other hand, several studies reported higher amounts of acetate in ASD from urine and faecal samples [48,66,70]. Similarly, butyrate is highly involved in the energy metabolism in the gut since it is the preferred carbon source for colonocytes. Butyrate-producing bacteria mainly belong to the Firmicutes phylum, more specifically to *Lachnospiraceae* and *Ruminococcaceae* families [94]. Besides interacting with certain host receptors modulating immune function, butyrate has a great impact on epigenetic modifications because of its ability to inhibit histone deacetylases (HDACs) [80]. Epigenetic changes may not only lead to local events but systemic. These epigenetic modifications alter the host’s gene expression and have been linked to processes such as apoptosis, inflammation and lipid metabolism [81]. SCFA analyses of faecal and caecal samples from individuals with ASD showed higher amounts of butyrate compared to control groups [44,49,58,70], although opposite results were also observed [17,41]. Propionate has been associated with multiple health-promoting effects for humans [62], although its administration via intracerebroventricular injection has been shown to induce autistic-like symptoms in rats [100]. It is important to mention that the concentration of propionate used in the experiment largely exceeds the physiological concentrations found in normal conditions [96]. Propionate is synthesised from both carbohydrates and peptides by bacteria from Bacteroidetes phylum (including *Bacteroides* and *Prevotella*) and from Firmicutes phylum (including *Roseburia*, *Blautia* and *Coprococcus*), among others [101]. Propionate influences several molecular processes affecting intestinal homeostasis, catecholamine production in the brain, and immune and mitochondrial functions [102,103]. The analysis of SCFAs from faeces and caecum of individuals affected by ASD has also reported contrary outcomes showing to be either increased [48,70] or decreased [17,41] compared to control individuals.

Altogether, excessive amounts of SCFAs like propionate affect a multitude of molecular processes including signalling processes, epigenetic changes, barrier integrity, immune function and neurotransmitter synthesis and release [104,105]. Another fundamental role of SCFAs in the host’s function is the ability to modulate HDAC activity. Inhibition of HDACs targeted by SCFAs leads to epigenetic changes that may impact ASD development [106]. For example, one of the genes affected is CREB, an important transcriptional factor involved in neurodevelopmental processes and brain function [107]. Some of the molecular pathways that were reported to be altered due to SCFA-mediated epigenetic changes include neurotransmitter synthesis, immune activation and mitochondrial function [102]. 

Although the research on the role of SCFAs in host health has been thoroughly investigated and there are relevant data, there is little consensus on the role of SCFAs in neurological diseases such as ASD. Besides the production of SCFAs in the colon, which contains the largest microbial population within the gut, other important bioactive metabolites such as succinate, pyruvate or lactate are produced from carbohydrate metabolism [108]. For example, excessive succinate concentrations showed to be associated with an increase in oxidative stress and proinflammatory immune response via systemic interleukin 1 β (IL-1β) production. It was shown that an increase in succinate oxidation promotes the proinflammatory phenotype of macrophages that results in the production of proinflammatory cytokines [109]. This evidence is relevant for ASD as oxidative stress and inflammation were often described in individuals with ASD, in addition to increased levels of succinate detected in urine and blood samples from individuals affected by ASD [66,71,74]. Altogether, it is important to understand the dynamic relationship between the microbes that our gut harbour and their metabolic capacity based on carbon source availability.

### 5.2. Bacterial Metabolites from (Aromatic) Amino Acid Metabolism and Neurotransmitters

In the distal colon, numerous bacteria have the ability to ferment proteins and amino acids derived from diet and protein turnover which are essential for their growth [110]. Bacterial communities in the intestine influence the bioavailability and utilisation of certain amino acid-derived metabolites that have an impact on the host’s metabolic function [111]. Proteolytic fermentation by gut microbiota results in the production of branched short-chain fatty acids (BCFA), ammonia, poly- and mono-amines, indoles, phenols and sulphides, among others. Additionally, complex plant secondary metabolites can be transformed by gut bacteria into other phenolic compounds such as polyphenols, known as health-promoting metabolites [112]. The most studied pathways are related to aromatic amino acid metabolism (phenylalanine, tryptophan and tyrosine), whose intermediate and final products were found in the systemic circulation [113]. Concentrations of free phenylalanine, tyrosine and tryptophan were shown to be increased in faecal samples from individuals diagnosed with ASD compared to healthy individuals [41,48]. However, Wang et al. (2019) reported decreased faecal concentrations of phenylalanine and tyrosine [42]. Contrary to bacterial-derived compounds from complex carbohydrates, protein-fermented metabolites are associated with detrimental effects on the host [114]. Overall, these results indicate abnormal availability of aromatic amino acids in the intestine of individuals with ASD and it can also mean that systemic concentrations of these amino acids, and their derivatives, are altered possibly due to gut dysbiosis and increased intestinal barrier permeability. 

Multiple compounds from tyrosine and phenylalanine-derived bacterial metabolism were shown to be at abnormal concentrations in individuals affected by ASD compared to healthy controls. In particular, tyrosine-derived *para*-cresol (p-cresol; 4-methylphenol) levels are higher in both urinary and faecal samples from children with ASD [41,48,67,73,80]. p-Cresol is known to be produced by several species belonging to the *Clostridia* class [115]. It is transformed by the host into p-cresyl sulfate for further excretion via urine. Some of the molecular processes affected by this metabolite include DNA damage, apoptosis induction, and inflammation [116]. p-Cresol is one of the major protein-bound uremic toxins and its accumulation in serum is considered to be a biomarker for chronic kidney disease indicating chronic inflammation and endothelial barrier dysfunction [117]. Interestingly, it was also hypothesised that p-cresyl sulfate is implicated in neurological disorders. In a recent study, p-cresyl sulfate concentration showed to be increased in cerebrospinal fluid; also, p-cresyl sulfate cerebrospinal fluid/plasma ratio was observed to be higher in individuals with Parkinson’s disease compared to control individuals, suggesting to influence the progression of the disease [118]. One of the physiological activities of p-cresyl sulfate is to induce oxidative stress, thus circulating p-cresyl sulfate that reaches and crosses the BBB and may promote an inflammatory environment in the brain [119]. Another common problem in individuals with ASD is the loss of effectively detoxifying compounds via sulfonation in the liver. Some of these compounds comprise hormones, neurotransmitters and bacterial-derived metabolites such as p-cresol which can influence the CNS [120,121]. These are some proposed mechanisms of how microbiota-derived metabolites may influence ASD pathogenesis. 

Dopamine, norepinephrine and epinephrine are derived products from the metabolism of phenylalanine [65]. In a recent study, data showed lower levels of norepinephrine, epinephrine and higher levels of dopamine and p-cresol in the urine of autistic children compared to healthy individuals [80]. These results can be explained as an effect of p-cresol, which inhibits dopamine β-hydroxylase, an essential enzyme that converts dopamine into norepinephrine [80]. Another study showed that p-cresol-treated mice developed social impairments and presented lower dopamine activity, these parameters were restored after faecal microbial transplantation from control mice. On the other hand, these effects were transferred by faecal microbial transplantation of p-cresol-treated mice into control mice suggesting the involvement of a microbiota-dependent mechanism [122]. Additionally, it was shown that dopamine concentrations were higher in certain brain regions, such as the nucleus accumbens, the caudate-putamen and the amygdala (prefrontal cortex and hippocampus did not show significant differences) from BTBR T + tf/J mice (model for ASD) after p-cresol administration [123]. Although in a previous study, dopamine concentration was significantly decreased in the prefrontal cortex collected from a cow’s milk allergy murine model with ASD-like behavioural phenotype. The concentration of 3,4-dihydroxyphenylacetic acid (DOPAC; a dopamine-derived metabolite) was significantly enhanced in the amygdala of cow milk allergic mice compared to control mice, and dopamine followed a similar increasing trend [124]. The prefrontal cortex was described as an inhibitory regulator of the dopaminergic pathway in the nucleus accumbens after amygdala activation [125]. The mesocorticolimbic circuit comprises neuronal interaction between the prefrontal cortex and nucleus accumbens, and it was hypothesised that dysfunctional dopaminergic pathway in these areas contributes to cognitive abnormalities, social interaction and communication deficits [126,127]. Dopamine cannot cross the BBB in normal conditions but it is known that gut microbiota can influence dopamine release in the brain via vagal nerve signalling [128]. The altered dopaminergic system may be linked to the development of autistic symptoms such as stereotyped behaviours, hyperactivity and anxiety [24]. Considering these findings, it is possible that an imbalance of dopaminergic processes in the brain may impact behavioural and cognitive functions in ASD.

Several metabolites produced in multiple steps, including bacterial and host metabolites of phenylalanine, were found to be increased in the urine of autistic children. Some of these metabolites were phenylpropionic acid and its derivative, 3-(3 hydroxyphenyl)-3 hydroxypropionic acid (HPHPA) [65,68,77]. In addition, Shaw et al. (2010) reported that HPHPA amounts drastically decreased in the urine of individuals with ASD after antibiotic treatment, presumably due to a decreased population of *Clostridia* species (main producers of phenylpropionic acid) [65]. It is known that phenylpropionic acid, and probably HPHPA because of structural similitude, inhibit enkephalinases that lead to an elevation of enkephalins in the brain. Enkephalins together with β-endorphins (produced in the pituitary gland) are reported as endogenous opioids modulating brain functions including pain, and social and stereotypical behaviour [129,130]. β-endorphin levels were found to be increased in plasma in ASD children [131]. According to it, this study suggested a higher susceptibility to acute stress response in autistic individuals, although circulating β-endorphins do not resemble levels in the brain as they cannot cross the BBB. Moreover, high amounts of HPHPA produced by gut bacteria may lead to a depletion of catecholamines, hormones produced by the adrenal glands (such as norepinephrine, epinephrine and dopamine), due to substrate competition for dopamine β-hydroxylase [24,132]. 

**Figure 2:**
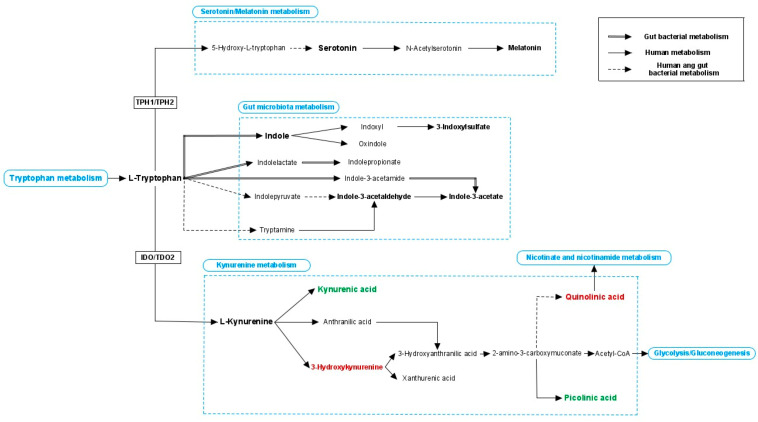
Tryptophan metabolism. Tryptophan can enter three different pathways: serotonin/melatonin pathway, gut microbial metabolism and kynurenine pathway. Tryptophan hydrolase 1 and 2 (TPH1 and TPH2, which are expressed in the intestine and brain respectively) enzymes are key regulators of serotonin production. Many indolic compounds are derived from bacterial metabolism but also human enzymes contribute to their production, and they showed to affect multiple processes of the host. Indoleamine-2,3-dioxygenase (IDO, expressed in different organs) and tryptophan-2,3-dioxygenase (TDO2, expressed specifically in the liver) control the first and rate-limiting step of the kynurenine metabolism. Kynurenine can be metabolised into kynurenic acid that is considered to have neuroprotective properties. Alternatively, kynurenine can enter another pathway where several compounds were reported to have neurotoxic effects. Continue line: human metabolism; double line: bacterial metabolism; discontinue line; both human and bacterial metabolism; green coloured word: neuroprotective effects; red coloured word: neurotoxic effects.

Tryptophan can enter the three following metabolic pathways: (i) serotonin pathway, (ii) kynurenine pathway, and (iii) gut bacterial metabolism [108], see Figure 2. Serotonin modulates multiple intestinal- and neural-related functions including intestinal motility, nutrients’ secretion and absorption, appetite, mood, verbal ability and cognitive function, among others [130,133,134]. The vast majority of serotonin synthesis occurs in the gut through the tryptophan hydrolase 1 enzyme (TPH1); therefore, brain serotonin (produced through the tryptophan hydrolase 2 enzyme, TPH2) does not seem to have a great impact on blood levels of serotonin. However, a recent study demonstrated that elevated concentrations of brain serotonin contribute to higher levels of systemic serotonin [135]. Being mostly synthesised in the gut, serotonin production is highly influenced by intestinal microbes. It was demonstrated that spore-forming bacteria are associated with elevated levels of serotonin in the colon and blood by activating its production by enterochromaffin cells [136]. Furthermore, the gut microbiota is essential for ENS maturation and neuronal serotonin release in the myenteric plexus [137]. Since serotonin is a regulator of intestinal function, it may also affect the microbial species that are able to colonise the gut. The majority of studies of neurotransmitter dysfunction in ASD pathophysiology have targeted the serotonergic system since autistic individuals suffer from hyperserotonaemia (high concentration of serotonin in the blood) [138,139]. In a murine model for ASD, serotonin levels in the brain were assessed and an increase in serotonin was observed in the caudate-putamen compared to control mice [123]. Although hyperserotonaemia is mainly associated with genetic problems involving the serotonin transporter gene [140], gut microbiota also influence serotonin production. Certain bacteria are able to directly synthesise serotonin, while others may indirectly influence its production via epigenetic-mediated stimulation of the expression of the *Tph1* gene in the intestine [141]. Additionally, SCFAs promote serotonin synthesis in enterochromaffin cells by stimulating *Tph1* expression [141]. Some studies have explored the concentration of serotonin in the gut of mouse models of ASD and concluded that it was significantly lower compared to healthy mice [44,142]. 

Gut microbiota plays an important role in regulating the availability of circulating tryptophan [143]. In particular, tryptophan metabolism is a key modulator of behavioural functions such as mood, anxiety and stress. Most of the available tryptophan (90%) in the intestine is metabolically transformed via the kynurenine pathway. Inflammation and stress contribute to the upregulation of enzymatic genes involved in kynurenine production (indoleamine-2,3-dioxygenase; IDO1 expressed in all tissues, and tryptophan-2,3-dioxygenase; TDO mainly expressed in the liver) [144]. Recently, kynurenine metabolism has become an area of study in neurological diseases with a possible neuroinflammatory component [145,146]. Muramaki et al.’s (2019) study indicates that kynurenine metabolism is altered in a murine model for ASD [147]. Serum samples showed greater quantities of kynurenine, kynurenic acid and 3-hydroxy kynurenine, while brain samples obtained from the frontal cortex showed higher concentrations of 3-hydroxy kynurenine and 3-hydroxy anthranilic acid. These last two mentioned metabolites belong to the neurotoxic branch that is often activated in reactive microglial cells and are converted into quinolinic acid [147]. Besides regulating levels of circulating tryptophan, intestinal microbes also may influence the kynurenine pathway by controlling the maturation and function of microglial cells [148]. Moreover, the involvement of the kynurenine pathway in the disease was also investigated in autistic children showing lower amounts of neuroprotective metabolites in serum, such as picolinic acid. They also showed to have a higher amount of neurotoxic metabolites, such as quinolinic acid, in comparison to healthy children [75]. Additionally, peripheral kynurenine and kynurenine/tryptophan ratios were increased in children diagnosed with ASD, indicating a reduction of tryptophan availability and an impaired serotonergic system that might lead to behavioural abnormalities [75]. 

In ASD, increased BBB permeability, which has been related to gut dysbiosis, possibly allows the pass of kynurenine-derived metabolites such as kynurenic acid and quinolinic acid. These two compounds are an antagonist and an agonist of the N-methyl-D-aspartate receptor (NMDAR), respectively [143]. The NMDAR is involved in the proper development of CNS, cognitive function and stereotypic behaviour, among others. Abnormal NMDAR-mediated responses were described in ASD and its potential as a therapeutic target was considered [149]. Additionally, lower levels of brain-derived neurotrophic factor (BDNF), a neurotrophin involved in neural development and function, were reported in ASD. Intestinal microbes have proven to activate *Bdnf* expression in the brain [21]. BDNF and NMDAR are closely involved in certain neuronal processes, thus abnormal levels may contribute to ASD pathogenesis as was shown in other neurological disorders such as schizophrenia and depression [22,150]. Aryl hydrocarbon receptor (AhR) can be activated by gut microbiota-derived metabolites, specifically indolic compounds. One of its roles is regulating the expression of the enzymes TDO and IDO1, which catalyse the first and rate-limiting step of the catabolism of the amino acid tryptophan in the kynurenine pathway. In addition, some bacteria are able to directly produce kynurenine and others can produce derivative metabolites [151]. Furthermore, kynurenine metabolism influences the host’s energy metabolism and mitochondrial function through the synthesis of nicotinamide adenine dinucleotide and its reduced form (NAD+/NADH). Quinolinic acid is the precursor molecule of NAD+ involved in multiple physiological processes that take place in the human body [152]. Balanced levels of NAD+/NADH are essential to maintain redox homeostasis and abnormal nicotinic metabolism is present in several neuroinflammatory diseases [153], including ASD [154].

Besides the serotonin and kynurenine pathways, tryptophan can be converted into multiple indolic compounds by intestinal bacteria [155]. Indole and some of its derivatives such as indole-3-propionate and indole-3-aldehyde are associated with promoting the host’s health via the activation of AhR which stimulates Th17 and intraepithelial lymphocytes [156]. Moreover, they can activate pregnane X receptors (PXR) which are involved in mucosal immune homeostasis and intestinal barrier function [157]. In ASD, indole-3-acetate concentration is demonstrated to be reduced in urine and faecal samples compared to healthy individuals [43,71]. This metabolite shares structural similarities with indole-3-propionate; thus, it was proposed to exert similar beneficial effects on the host. Therefore, decreased levels of indole-3-acetate may be translated in loss of intestinal and immune homeostasis. Other indolic compounds are considered to be uremic toxins and were proposed to be a link between microbiota dysbiosis and renal disease [158]. Uremic toxins not solely impact renal function, but also neurological function, contributing to certain brain disorders [159]. For example, bacterial-produced indole is associated with anxiety-like behaviour in rats. The authors of this report suggested that the activation of the vagus nerve and/or an imbalance in tryptophan metabolism were responsible for the behavioural abnormalities [160]. Indole, produced by a wide variety of bacteria including *Escherichia* and *Clostridium* species, is converted into 3-indoxylsulfate in the liver to be further excreted in urine [161]. The metabolite, 3-Indoxylsulfate, is believed to cause detrimental effects on the host, and it has been frequently associated with oxidative stress, cardiovascular and renal diseases [116]. In chronic kidney disease, elevated levels of 3-indoxylsulfate in serum samples showed a strong correlation with cognitive impairment and intestinal epithelial barrier disruption [162,163]. Olesova et al. (2020) investigated specific uremic toxins in children affected by ASD. The metabolite, 3-Indoxylsulfate, was drastically increased in the subset of autistic children older than 6 years old compared to healthy children (no differences were seen in the subset of autistic children younger than 6 years old) [82]. In addition, indole can be metabolised into oxindole and conjugates in the liver and these compounds are suggested to have neurodepressant activity [164]. However, further research is warranted in order to better explore bacterial activity in the gut and to elucidate which microbial species are responsible of the production of these metabolites depending on energy source availability.

Abnormal glutamate metabolism leads to neural dysfunction and seems to play an important role in ASD pathogenesis. Glutamate is tightly involved in neurotransmission and redox processes. γ-Amino butyric acid (GABA, produced from glutamate) and glutamate are responsible for inhibitory/excitatory signalling leading to brain dysfunction [165]. Regarding the levels of glutamate in ASD, controversial results have been published. Some studies reported higher concentrations of glutamate in faeces, urine and plasma from children suffering from autism [48,74,80], while other investigations showed lesser amounts in faeces and urine [41,66] compared to healthy individuals. Interestingly, de Angelis et al. (2013) who identified higher quantities of glutamate in faeces from autistic children, additionally reported fewer quantities of GABA [48]. These data differ from the study of Kang et al. (2018) who identified a lower amount of both glutamate and GABA in faecal samples from autistic children [41]. Moreover, a strong correlation between glutamate levels and microbial composition was found in faecal samples from individuals with ASD [42]. Microbiota can play an important role in glutamate/GABA metabolism by being able to metabolise both compounds [166]. It is evident that alterations in glutamate and GABA processes in the intestine are common in ASD; nonetheless, the lack of consistent results and unclear mechanism of action in ASD pathophysiology hinders its use as a biomarker for the disease. Glutamine is fundamental for glutamate synthesis and, thus, GABA in the brain. Interestingly, the investigation of the glutamate–glutamine cycle in ASD revealed that it is impaired in comparison to a healthy state [167].

Moreover, glutamate is a precursor of glutathione (GSH) whose main function is to regulate redox metabolism acting as an antioxidant compound in the host. GSH is converted into glutathione disulfide (GSSG) that in turn can be reduced to GSH. In the brain, its pivotal role counteracts the excess of reactive oxygen species (ROS) and therefore avoids the alteration of other host processes such as neuronal damage and loss [168]. GSH insufficiency was reported in several neurodevelopmental and neurodegenerative disorders, suggesting its important role in the onset and progression of the diseases and its potential use as a therapeutic target [169]. Several human studies have described redox metabolism abnormalities in autism. GSH concentrations and GSH/GSSG ratios were significantly lower in plasma and brain samples from individuals affected by ASD compared to healthy controls. In addition, Rose et al. (2012) and Frye et al. (2013) investigated the amount of 3-clorotyrosine, a biomarker for inflammation, that resulted to be elevated in plasma and brain of autistic children [64,69,72]. Gut microbiota influences redox homeostasis in the gut as microbes produce and compete with the host for electron acceptor compounds [170]. Thus, it would be logical to hypothesise that there is an associative relationship between gut dysbiosis and systemic redox dysfunction. These pieces of evidence indicate that an imbalanced redox metabolism is often present as a comorbid condition at least in a subset of autistic children. Besides its role in neural function, GSH balance has a great impact on mitochondrial oxidative levels, when impaired it was also linked to contributing to ASD pathophysiology. 

### 5.3. Other Bacterial Metabolites 

Bile acid metabolism is involved in health and disease considering that it is essential for immune function homeostasis and lipid metabolism [108]. Primary bile acids are produced from cholesterol by the liver, then excreted into the proximal small intestine promoting fat digestion among other functions. Most primary bile acids are reabsorbed in the distal ileum, the ones that remain in the gastrointestinal tract enter the colon where some bacteria can utilise them to produce secondary bile acids [171]. Gut dysbiosis has been linked to abnormal amounts of secondary bile acids in intestinal disorders such as inflammatory bowel disease and colorectal cancer, but also in multiple brain-related medical conditions, such as autism [44,101,172]. The molecular mechanisms by which secondary bile acids can influence ASD are the following: (i) increased barrier permeability due to the high hydrophobic profile of secondary bile acids, (ii) influence in neurotransmitter signalling as serotonin, and (iii) immune dysfunction. In an animal model for ASD, researchers found a strong correlation between an impaired bacteria-mediated bile acid transformation and a reduction in serotonin production [44]. Additionally, bacterial-derived bile acids have a big impact on the host’s gene expression via the interaction with receptors such as PXR and the G protein-coupled receptor TGCR5. This leads to the activation of multiple signalling cascades that result in gene expression changes including genes involved in inflammation [173].

Gut bacteria also alter the metabolism of fatty acids, phospholipids and vitamins mediated by the production of secondary bile acids. Opposed to T helper cells (Th1, Th2 and Th17) that obtain energy from glucose metabolism, T regulatory cells rely on fatty acid oxidation [174]. Abnormal host’s expression of genes involved in lipid metabolisms, such as *acc1* and *fas*, is mediated by gut microbiota [175]. Moreover, differences in phospholipid profiles were found in autistic individuals compared to healthy subjects. Phospholipids have a great impact on a plethora of functions within the host, including the structure of physiological barriers and cellular membranes, signal transduction and antioxidant properties [100]. Altered vitamin levels were also reported in autism, being vitamin B12 and vitamin D deficiencies the most frequent among individuals with ASD [176]. Specifically, vitamin B12 and vitamin D are key molecules to maintain gut homeostasis. Even though some bacteria can produce these vitamins, the gut microbiota is shaped by their availabilities [177,178]. It is possible that these differences promote an overactivation of the immune system, gut dysbiosis, oxidative damage as well as neuronal dysfunction. Moreover, gut microbiota may be involved in mitochondrial function and redox homeostasis by influencing the redox potential and the ratio of lactate/pyruvate in the human body. Increased numbers of propionate-producing bacteria showed to impair the tricarboxylic acid cycle compromising redox homeostasis [72]. Overall, evidence indicates that gut microbiota clearly impacts neurochemistry and neurological function.

## 6. Future Perspectives and Conclusions—Shaping the Gut Microbiota

To date, there is no specific treatment designed for ASD and the pathophysiology is still unclear. Current evidence indicates that the gut microbiome can trigger and/or exacerbate the disease through bacterial metabolite production. Therefore, a better understanding of the microbiome-related metabolites and their role in the gut–brain axis might bring new insights into ASD pathology progression, as well as lead to new therapeutic approaches. 

More specifically, promoting a “healthy” gut microbiota might be a promising avenue for improving ASD symptomatology. Recent studies using faecal microbiota transplantation or dietary interventions for ASD treatment showed promising results [179]. Kang et al. (2017, 2019 and 2020) demonstrated that faecal microbiota transplantation in children with ASD (i) improved gastrointestinal symptoms and ASD-related symptoms, (ii) led to the more diverse gut microbiota, and (iii) restored the level of several plasma metabolites [180,181,182], suggesting it to be a favourable tool for ameliorating the symptoms of a subset of individuals with ASD and intestinal comorbidities. However, there are some limitations as sample size, and more studies are needed to explore the potential therapeutic role of faecal microbiota transplantation in ASD. Both human and animal studies of probiotic supplementation in ASD have shown to ameliorate not only intestinal-related symptoms but also ASD-related core symptoms [56,60,183]. Similarly, Grimaldi et al. (2018) observed improvements in social behaviour and significant changes in gut microbiota composition after prebiotic administration [184]. Nevertheless, a major limitation of these studies is our lack of understanding of the exact working mechanism of these pro- and pre-biotics and whether changes in specific bacterial metabolites, such as increases in possible beneficial SCFAs, are involved in the reduction of intestinal and/or behavioral problems. Again, pointing out whether changes in gut microbiota composition and function are a consequence of ASD and perpetuate disease development or whether they are the initial trigger. This highlights the urgent need for a mechanistic and predictive understanding of microbiome function. 

The first step towards this goal is understanding the mechanisms that drive microbiome composition and function, including the interactions of species with the environment (the diet) and with each other, which are still poorly understood. Recent experiments are showing the critical role that metabolic traits play in the assembly of microbial communities and the maintenance of their diversity [185,186,187]. These results suggest that a quantitative and predictive understanding of the microbiome’s metabolic structure and function is within reach, and will likely require an interdisciplinary combination of experiments, theory and computational modelling [188]. Particularly promising are genome-scale metabolic modelling methods, which are able to predict with fair accuracy metabolic phenotypes (e.g., growth or metabolite secretion) from genome data [189]. These models are already being used to design interventions in important microbial communities [190], illustrating their potential both for mechanistically understanding microbial interactions and as a tool for rational manipulation.

In particular, new metabolic modelling platforms such as COMETS (Computation of Microbial Ecosystems in Time and Space) allow the simulation of microbial communities with thousands of species in ecologically realistic settings, e.g., mimicking natural environments such as the gut [191]. This paves the way towards the important milestone of building realistic in silico models of the gut microbiome from sequence data, possibly even in a personalised way. The availability of such models could allow us to quickly and inexpensively test treatments targeting the microbiome, including dietary interventions, reducing the time and cost of experimental and clinical studies.

Many gut bacteria-derived metabolites are altered in ASD; however, more in-depth studies are essential to demonstrate the (causative) role of these metabolites in ASD. Besides the research centred on the gut microbiome and its function, a second, important line of research will require us to understand precisely how microbiome-derived metabolites interact with the host. More specifically, we need to understand how it compromises the maintenance of intestinal homeostasis, and how it contributes to neurodevelopmental disorder susceptibility. Overall, achieving the goals of understanding the composition and function of the gut microbiome and its interaction with our own body holds great promise for a successful design of treatment for ASD and might allow us to identify specific biomarkers for improving diagnosis and identifying new therapeutic targets.

## Supplementary Materials

The following are available online at https://www.mdpi.com/article/10.3390/ijms221810052/s1.
